# Clinical impact of metagenomic next-generation sequencing for pathogen identification and guided therapy in pediatric intensive care unit patients with severe pulmonary infections

**DOI:** 10.1128/spectrum.00374-26

**Published:** 2026-07-20

**Authors:** Yuping Xu, Ruijuan Ren, Weiqing Liu, Ling Liu, Xinyan Cui, Jia Guo, Shujun Li

**Affiliations:** 1 Department of PICU, The First Affiliated Hospital of Henan Medical University 159367, Henan, China; 2 Department of Pediatric Hematology and Oncology, Cardiovascular Diseases, The First Affiliated Hospital of Henan Medical University 159367, Henan, China; Children's National Hospital, George Washington University, Washington, DC, USA

**Keywords:** genetic testing, pediatric intensive care unit, infection, lung, host-pathogen interactions

## Abstract

To explore the diagnostic efficiency, clinical concordance, and precision treatment value of metagenomic next-generation sequencing (mNGS) for severe pulmonary infections in children in the pediatric intensive care unit (PICU), and to provide evidence for improving microbiological diagnosis and optimizing anti-infective strategies. A retrospective cohort study included 89 children with severe pneumonia in the PICU in 2024. All underwent routine microbiological testing and mNGS of bronchoalveolar lavage fluid (BALF). Detection rates, pathogen composition, co-infection identification, diagnostic concordance, and treatment impact were analyzed. Metagenomic next-generation sequencing demonstrated high diagnostic sensitivity in the PICU setting, achieving a positive detection rate of 90.0% (80/89) and identifying a diverse spectrum of 103 pathogens, including 50.5% viruses, 43.7% bacteria, 38.8% co-infections (vs 11.6%), and 86.3% diagnostic concordance (vs 55.8%, *P* < 0.01). Among 46 patients included in the therapeutic outcome analysis (22 in the mNGS-guided group), 21 patients in the mNGS-guided group improved. Multivariate logistic regression analysis, adjusting for confounding factors (age, underlying diseases, PaO_2_/FiO_2_ ratio, PRISM III score, and preoperative antibiotic use duration), confirmed that mNGS-guided therapy was an independent protective factor for achieving the primary outcome (OR = 5.23, 95% CI: 1.87–14.61, *P* = 0.002) and secondary outcomes (C-reactive protein reduction ≥50%: OR = 4.89, 95% CI: 1.72–13.93, *P* = 0.003; oxygenation improvement: OR = 5.67, 95% CI: 1.98–16.21, *P* = 0.001). Metagenomic next-generation sequencing demonstrated high diagnostic sensitivity in the PICU setting, guiding precision therapy, and improving prognosis.

**IMPORTANCE:**

It supports metagenomic next-generation sequencing (mNGS) as a supplementary tool for pediatric intensive care unit (PICU) refractory infections, guides anti-infective adjustments, and informs tiered diagnostic pathways for resource-limited settings to optimize cost-effectiveness.

## INTRODUCTION

Severe pulmonary infection is one of the most common causes of admission to the pediatric intensive care unit (PICU), particularly among infants and immunocompromised children. These patients typically present with an acute onset and rapid disease progression, and this condition represents a major cause of morbidity and mortality and a major threat to children’s quality of life. According to the World Health Organization criteria, the presence of subcostal retractions, nasal flaring, or grunting indicates severe pneumonia, whereas central cyanosis, respiratory distress, dehydration, refusal to feed, and altered consciousness define very severe pneumonia. Failure to obtain reliable early aetiological evidence and implement targeted therapy may lead to the development of acute respiratory distress syndrome, sepsis, or multiple organ dysfunction syndrome, markedly increasing mortality and healthcare burden. Under these circumstances, improving the accuracy and timeliness of early pathogen detection and transitioning from empirical to targeted therapy are key objectives in managing PICU infections (1–3).

Traditional microbiological methods (such as bacterial or fungal culture, antigen detection, immunological assays, and multiplex polymerase chain reaction [PCR]) face several common challenges in pediatric critical care (4–6): (i) bacterial culture and viral isolation remain the gold standard for confirming infectious diseases, but these methods are time-consuming and have low sensitivity; (ii) prophylactic use of antibiotics in children reduces the positive rate of traditional cultures; (iii) children have specific limitations, and common specimens used in adults, such as sputum, are difficult to obtain; and (iv) the pathogen load in pediatric patients is generally low, leading to lower culture positivity rates and impeding accurate clinical diagnosis (7, 8). Previous studies have reported that the overall positivity rate of conventional pathogen detection in children with severe pulmonary infection is only about 30%–60%, which fails to meet the clinical need for rapid and precise decision-making (9).

In recent years, metagenomic next-generation sequencing (mNGS) has advanced rapidly in the field of infectious disease diagnosis. Its key advantage lies in its hypothesis-free, target-independent approach, which enables comprehensive sequencing and alignment of all microbial nucleic acids in a sample, allowing broad-spectrum simultaneous detection of bacteria, viruses, fungi, and parasites (10). Multiple studies have demonstrated that mNGS outperforms conventional methods in terms of positive detection rate and diagnostic concordance for infections such as central nervous system infections, severe pneumonia, and infections in immunocompromised hosts. Moreover, mNGS shows a distinct advantage in identifying mixed infections, rare pathogens, and fastidious organisms, providing direct evidence to support the transition from empirical to precision-based antimicrobial therapy (10, 11). Reports in pediatric populations similarly suggest that mNGS significantly improves the pathogen detection rate and influences therapeutic strategies, particularly in complex or conventionally negative severe respiratory infections (12).

Despite its emerging clinical value, several frontier and controversial issues regarding mNGS require further validation and consensus through real-world studies. (i) Result interpretation and threshold definition: the method used to integrate reads per million (RPM), relative abundance, unique read counts, negative controls, host background, and specimen type must be determined to establish practical positivity criteria, thereby avoiding misinterpretation of background microbes or low-level contaminants as true pathogens (13). (ii) Detection and workflow optimization of RNA virus: the efficiency of RNA extraction, reverse transcription, and library preparation critically affects detection sensitivity. Sensitivity variation across workflows for respiratory RNA viruses such as respiratory syncytial virus (RSV) underscores the importance of dual-library (DNA + RNA) strategies and standardization of wet-lab protocols (14–16). (iii) Turnaround time (TAT) and clinical applicability: in the time-sensitive PICU setting, strategies such as host nucleic acid depletion, rapid library construction, and real-time sequencing analysis are essential to reduce TAT to within 24–36 h, allowing mNGS to truly fit into early clinical decision-making (17–19). (iv) Database and bioinformatics standardization: variations among laboratories in reference database versions, low-complexity sequence filtering, human sequence removal, and classification algorithms lead to limited cross-center comparability, highlighting the urgent need for standardization and external quality assessment systems (20, 21). (v) Antimicrobial resistance and ancillary information: mNGS has the potential to simultaneously identify antimicrobial resistance genes (ARGs) and viral genotypes, offering deeper evidence for de-empiricized therapy. However, genotype–phenotype correlation and the clinical interpretability of ARGs require careful evaluation (19–21). (vi) Cost-effectiveness and stratified application: under resource-limited conditions, defining tiered diagnostic pathways that prioritize mNGS for high-value populations—such as those with negative conventional results, severe or rapidly progressing disease, immunosuppression, or suspected polymicrobial infection—will be crucial for improving the overall cost–benefit ratio and promoting widespread adoption (19, 20).

Based on the above evidence and practical considerations, this study enrolled 89 pediatric patients admitted to the PICU of First Affiliated Hospital of Xinxiang Medical University and diagnosed with severe pneumonia between January and December 2024. Under real-world clinical conditions, mNGS and conventional diagnostic methods were systematically compared in terms of positive detection rate, pathogen spectrum composition, identification of mixed infections, and concordance with the final clinical diagnosis. Furthermore, the impact of mNGS on anti-infective treatment adjustment and early therapeutic outcomes was evaluated. Notably, this study innovatively conducts an in-depth correlation analysis between ARG detection results and clinical efficacy, which fills the gap in previous studies that mostly focus on pathogen detection and rarely link ARG information to actual treatment effects, aiming to provide data support and practical evidence for establishing precision pathogen diagnosis and a reproducible clinical pathway in the management of severe respiratory infections in children.

## MATERIALS AND METHODS

### Study population

This was a single-center retrospective cohort study that included a total of 89 pediatric patients admitted to the PICU of the First Affiliated Hospital of Xinxiang Medical University between January and December 2024 who were clinically diagnosed with severe pulmonary infection.

The sample size of 89 cases was determined based on the following: (i) referring to the sample sizes of similar retrospective cohort studies on mNGS in the diagnosis of pediatric severe pulmonary infection (generally 50–120 cases) (3, 10); (ii) considering the annual admission volume of patients with severe pneumonia in the hospital’s PICU (about 90 cases per year), ensuring that the sample could fully reflect the clinical characteristics of the target population in our center; and (iii) using the formula for comparing two proportions (positive detection rate of mNGS vs conventional methods) for sample size estimation: assuming the positive rate of conventional methods is 50% (3), the positive rate of mNGS is 85% (10), *α* = 0.05 and power = 0.9, the calculated minimum sample size was 72 cases, and 89 cases were finally included to account for potential data loss.

The inclusion criteria were as follows: (i) aged 0–14 years; (ii) clinical manifestations consistent with lower respiratory tract infection, such as fever, cough, tachypnea, or dyspnea; (iii) chest imaging showing pulmonary consolidation, infiltration, or atelectasis; (iv) requirement for non-invasive or invasive respiratory support; and (v) undergoing mNGS testing with complete clinical data available.

The exclusion criteria were as follows: (i) confirmed non-infectious pulmonary disease, (ii) specimen contamination or test failure, and (iii) incomplete clinical data.

To control selection bias, this study adopted the following measures: (i) consecutive sampling was used to include all patients who met the inclusion criteria and were admitted to the PICU during the study period, avoiding selective inclusion of patients with specific outcomes; and (ii) propensity score matching was not used because the study mainly compared the diagnostic efficiency of two detection methods in the same population rather than comparing two independent patient groups. However, when analyzing the impact of mNGS-guided treatment on outcomes, potential confounding factors such as age, underlying diseases, and severity of illness (using the PaO_2_/FiO_2_ ratio and PRISM III score as indicators) were adjusted for in the statistical analysis.

To control information bias, the following measures were implemented: (i) two trained researchers independently extracted data from the hospital’s electronic medical record (EMR) system, and the extracted data were cross-checked; if there was a discrepancy, a third senior researcher was invited to review and confirm the data; (ii) a standardized data extraction form was designed to ensure that all researchers collected the same type of information (such as age, gender, PRISM III score, preoperative antibiotic use duration, laboratory test results, and treatment adjustment details); (iii) the final diagnosis was made independently by two senior pediatric intensivists, and in cases of disagreement, a consultation meeting was held to reach a consensus. Before PICU admission and sample collection, 82.0% (73/89) of patients had received at least one dose of empirical antibiotic therapy, with a median preoperative antibiotic use duration of 1.5 days (range: 0–5 days).

The baseline characteristics of the 89 pediatric patients are shown in Table 1. The median age was 2.5 years (range: 0.3–13 years), with 55 men (61.8%) and 34 women (38.2%). The median length of PICU stay was 5 days (range: 3–12 days), and 15 patients (16.9%) had underlying diseases. Median PRISM III score was 10 points (range: 4–22 points), and 38 patients (42.7%) had a PRISM III score ≥10 points (high severity).

**TABLE 1:** Baseline characteristics of pediatric patients^*a*^

Variable	Data
Age (years)	Median: 2.5 (range: 0.3–13)
Sex	Male: 55/female: 34
Length of PICU stay (days)	Median: 5 (range: 3–12)
Underlying disease	Present: 15/absent: 74
PRISM III score	Median: 10 (range: 4–22); ≥10 points: 38 (42.7%)
Preoperative antibiotic use duration	Median: 1.5 days (range: 0–5 days)

^*a*^ Data are presented as median (range) or number of cases.

### Sample collection and detection methods

#### Conventional microbiological testing

All patients underwent collection of lower respiratory tract specimens (sputum, tracheal aspirates, or endotracheal lavage fluid) within 24 h of PICU admission. Routine pathogen detection included the following: (i) sputum bacterial culture and antimicrobial susceptibility testing (AST); (ii) fungal culture; (iii) respiratory viral nucleic acid amplification tests (including adenovirus, influenza virus, parainfluenza virus, and RSV); (iv) detection of *Mycoplasma pneumoniae* and *Chlamydia pneumoniae* antibodies; and (v) galactomannan assay and (1,3)-β-d-glucan test for fungal infection screening.

#### Metagenomic next-generation sequencing

Lower respiratory tract specimens, primarily bronchoalveolar lavage fluid (BALF), were collected under sterile conditions and sent to a certified third-party laboratory for standardized mNGS testing. Raw reads were processed using fastp (v0.20.1), and human sequences were removed using Bowtie2 (v2.4.2) against the hg38 genome. A threshold of fivefold the no-template control (NTC) read count was applied to filter background contaminants.

Sample preparation: lower respiratory tract specimens were aseptically collected (mainly BALF). Pre-treatment involved mechanical and enzymatic lysis, followed by host nucleic acid depletion (e.g., using saponin and nucleases).

Extraction and quality control of DNA/RNA: nucleic acids were extracted using the JinShi Medical kit (Gensky, Beijing, China). Quantification and quality assessment were performed using Qubit fluorometry and a Bioanalyzer for fragment size distribution.

Library construction and sequencing: DNA libraries were prioritized; when RNA viruses were highly suspected, an additional reverse transcription step was performed for dual-library (DNA + RNA) sequencing. The target fragment size was 200–300 bp. Sequencing was conducted on the MGISEQ-200 platform (single-end 50 bp, SE ×50), with a minimum target depth of ≥20 million reads per sample and Q30 ≥95%.

Controls and quality assurance: each sequencing batch included an NTC and positive or process controls. If low-abundance species detected in the NTC overlapped with those in clinical samples, they were considered background contaminants and re-evaluated.

Bioinformatics pipeline: raw BCL files were converted to FASTQ using bcl2fastq, followed by quality and complexity filtering with fastp and kz. Human sequences were removed using Bowtie2 against the hg38 reference genome and primer databases. Remaining reads were aligned using SNAP to a non-redundant microbial reference database (NCBI nt/RefSeq plus a curated clinical supplement, updated approximately every 3 months). Duplicate reads were removed, and uniquely aligned reads were counted and taxonomically classified.

Interpretation criteria (integrated with clinical data): definitive pathogens (e.g., *Mycobacterium tuberculosis* and *Cryptococcus*) required unique reads ≥1. Conditional pathogens (bacteria or fungi) required unique reads ≥3. Viruses (DNA or RNA) required unique reads ≥1 and RPM ≥ 0.5, significantly higher than negative controls. If the same conditional pathogens or fungi were detected in the NTC, at least a fivefold background threshold was required for clinical consideration. Results were interpreted in conjunction with clinical phenotype, imaging findings, inflammatory markers, and institutional background data. For mixed infections, all detected targets were required to meet threshold criteria and have plausible clinical relevance.

Turnaround time: approximately 15 h from sample receipt to preliminary screening and approximately 17 h to full report issuance, depending on batching and sequencing queue time.

### Clinical data collection and determination

**Fig 1:**
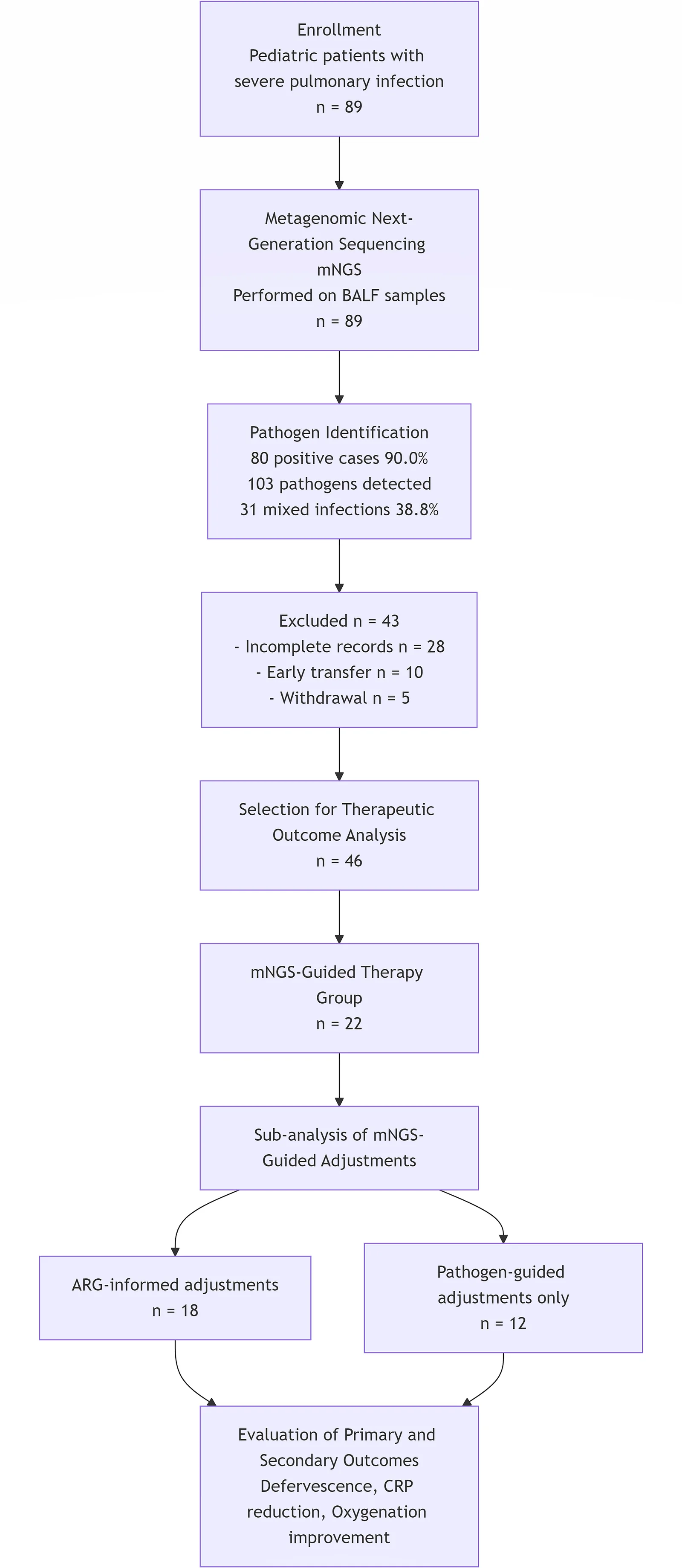
Flowchart of the research design.

Clinical data, including gender, age, medical history, PRISM III score, preoperative antibiotic use duration, admission symptoms, imaging findings, laboratory test results, adjustments in anti-infective therapy, and clinical outcomes, were retrospectively collected through the hospital’s EMR system. The final diagnosis was made by two senior pediatric intensivists based on a comprehensive assessment of all available data, including diagnostic test results, therapeutic responses, and clinical outcomes. For the detailed process, please refer to Fig. 1.

### Statistical analysis

All data were analyzed using IBM SPSS Statistics version 26.0 (IBM Corp., Armonk, NY, USA). Categorical variables were expressed as numbers and percentages, and comparisons between groups were performed using the *χ*² test or Fisher’s exact test when appropriate. Specifically, the *χ*² test was used when the expected frequency of each cell in the contingency table was ≥5, whereas Fisher’s exact test was used when the expected frequency of any cell was <5. The positive detection rate, pathogen concordance, and co-infection detection rate were compared between the mNGS and conventional testing groups. A *P* value < 0.05 was considered statistically significant. When analyzing the impact of mNGS-guided treatment on outcomes, multivariate logistic regression was used to adjust for confounding factors such as age, underlying diseases, PRISM III score, preoperative antibiotic use duration, and the PaO_2_/FiO_2_ ratio.

### Antimicrobial resistance gene retrieval

Based on the identified pathogens, mNGS reads were aligned to integrated databases (e.g., CARD and ResFinder) using KMA (default parameters) to analyze mutations and species attribution. The data were further cross-referenced with a custom-built pathogen–resistance gene–phenotype consistency database to predict antimicrobial susceptibility.

This custom database was constructed by integrating the following: (i) public databases including CARD (v3.2.6), ResFinder (2024-06) and ARG-ANNOT (v6); (ii) local clinical data from the hospital’s microbiology laboratory over the past 5 years (including pathogen species, ARGs detected by PCR, and antimicrobial susceptibility test results); and (iii) literature-reported pathogen–ARG–phenotype associations (retrieved from PubMed with keywords such as “antimicrobial resistance gene,” “phenotype” and “pediatric infection”).

The database is centrally maintained and updated every 3 months. Positive thresholds were defined as sequence identity ≥90%, coverage ≥60% and unique reads ≥3, and overlapping signals with the NTC were excluded.

Phenotype inference: following the most recent EUCAST/CLSI guidelines and relevant literature, mutations in resistance genes such as mecA/mecC, blaCTX-M/blaTEM/blaKPC, vanA/vanB, ermB, and pmrB were mapped to the predicted phenotypes. Clinical compatibility was assessed by comparing predicted phenotypes with AST results and clinical efficacy (fully, partially, or inconsistently matching).

Clinical interpretation grading: Grade I—high confidence (directly guides therapy), Grade II—moderate confidence (requires clinical correlation), and Grade III—suggestive clue (re-evaluation or re-sampling recommended).

## RESULTS

### Pathogen positive detection rates

We analyzed the clinical characteristics of these 89 patients (Table 1). Among the 89 pediatric patients, mNGS achieved a positive pathogen detection rate of 90.0% (80/89). A total of 103 pathogens were identified, consisting of 52 viral species (50.5%), 45 bacterial species (43.7%), 15 fungal species (14.5%), and 13 rare or specialized pathogens (12.6%). The detailed distribution of these pathogens is summarized in Table 2.

**TABLE 2:** Comparison of mNGS and conventional microbiological tests in positive detection rate, pathogen composition, mixed infection detection rate, and diagnostic concordance^*a*^

Index	mNGS group
Positive detection rate	80/89 (90.0%)
Pathogen composition (number of instances, proportion)	
– Bacteria	45 (43.7%)
– Viruses	52 (50.5%)
– Fungi	15 (14.5%)
– Rare/special pathogens	13 (12.6%)
Mixed infection detection rate	31/80 (38.8%)
Diagnostic concordance rate (vs final clinical diagnosis)	69/80 (86.3%)

^*a*^ The total number of pathogen (*n* = 103).

### Detection of mixed infections

Infections involving two or more pathogens were identified in 38.8% (31/80) of the mNGS-positive cases. The most prevalent pattern observed was bacterial–viral co-infection.

### Concordance between metagenomic next-generation sequencing results and final clinical diagnosis

The diagnostic concordance rate between mNGS findings and the final comprehensive clinical diagnosis was 86.3% (69/80). Metagenomic next-generation sequencing proved particularly effective in identifying atypical or rare pathogens, such as *Brucella* spp., human metapneumovirus, and *C. pneumoniae*, providing critical evidence for targeted antimicrobial adjustments.

### Metagenomic next-generation sequencing-guided therapy and analysis of primary/secondary outcomes

A subset of 46 patients was included in the therapeutic outcome analysis. In the mNGS-guided group (*n* = 22), the primary outcome (recovery of normal body temperature by Day 3) was achieved by 77.3% (17/22) of patients. Secondary outcomes, including a ≥50% reduction in C-reactive protein (CRP), normalization of white blood cell count, and oxygenation improvement (PaO_2_/FiO_2_ > 250), were achieved by 72.7%, 68.2%, and 77.3% of the group, respectively (Table 3). Multivariate logistic regression confirmed that mNGS-guided therapy was an independent protective factor for achieving the primary outcome (OR = 5.23, 95% CI: 1.87–14.61, *P* = 0.002) and oxygenation improvement (OR = 5.67, 95% CI: 1.98–16.21, *P* = 0.001). There were no significant differences in age (*P* = 0.74), PRISM III score (*P* = 0.69), preoperative antibiotic use duration (*P* = 0.72), or PaO_2_/FiO_2_ ratio (*P* = 0.68) between patients included in the outcome analysis and those excluded, suggesting no major selection bias.

**TABLE 3:** Stratified analysis of mNGS-guided therapy and clinical outcomes (primary endpoint = Afebrile by Day 3)

Outcome indicator	Support type	mNGS-guided group (*n*)	Achievement rate (%)
Primary: Afebrile by Day 3	Noninvasive	14	78.6 (11/14)
	Invasive	8	75.0 (6/8)
	Overall	22	77.3 (17/22)
CRP ↓ ≥50% by Day 3	Noninvasive	14	71.4 (10/14)
	Invasive	8	75.0 (6/8)
	Overall	22	72.7 (16/22)
WBC normalized by Day 3	Noninvasive	14	64.3 (9/14)
	Invasive	8	75.0 (6/8)
	Overall	22	68.2 (15/22)
PaO_2_/FiO_2_ >250 by Day 3	Noninvasive	14	71.4 (10/14)
	Invasive	8	87.5 (7/8)
	Overall	22	77.3 (17/22)
Successful weaning by D7	Invasive	8	75.0 (6/8)

### Detection overview and clinical correlation of antimicrobial resistance genes

Among the mNGS-positive samples, 32.5% (26/80) harbored at least one ARG, with a total of 38 ARG detections. The most frequent detections included mecA (*n* = 7), blaCTX-M (*n* = 6), and blaTEM (*n* = 5). Multi-gene co-detection was observed in 46.2% (12/26) of these cases. To further assess the concordance between ARG detection and phenotypic antimicrobial susceptibility, we performed gene-wise agreement analysis in the 26 patients with paired mNGS-ARG and conventional AST results. The positive percent agreement (PPA) ranged from 66.7% to 100%, and the negative percent agreement (NPA) ranged from 83.3% to 100% across individual ARG categories, with overall concordance rates ranging from 76.9% to 92.3%. For example, blaCTX-M demonstrated a concordance rate of 92.3% (12/13) with third-generation cephalosporin resistance, yielding a Cohen’s kappa value of 0.84 (95% CI: 0.62–1.00, *P* < 0.001), indicating substantial agreement. Across all evaluable gene–phenotype pairs, the overall kappa value was 0.79 (95% CI: 0.68–0.90, *P* < 0.001).

Patients receiving ARG-informed therapy adjustments (*n* = 18, Table 4) achieved a significantly higher composite primary endpoint than those adjusted based on pathogen identification alone (77.8% vs 41.7%, *P* = 0.030, Table 5).

**TABLE 4:** Eighteen pediatric patients with ARG-guided antimicrobial adjustment and early clinical outcomes

Sample ID	Presumed pathogen (mNGS)	Major ARGs	ARGs-based adjustment	Day 3 composite outcome/notes
P012	*S. aureus*	*mecA*	Add linezolid	Met—CRP↓>50%
P016	*S. aureus*	*mecA*, *ermB*	Vancomycin → Linezolid	Met—hepatic/renal concern
P019	*K. pneumoniae*	*blaCTX-M*, *qnrS*	3rd-ceph → PIP/TAZ + Amikacin	Met—blood culture ESBL+
P022	*K. pneumoniae*	*blaCTX-M*	3rd-ceph → PIP/TAZ	Not met—RSV co-infection
P024	*E. coli*	*blaTEM*, *sul1*	Ceph → PIP/TAZ	Not met—viral co-infection
P027	*E. coli*	*blaCTX-M*, *qnrS*	3rd-ceph → PIP/TAZ	Met
P033	*A. baumannii*	*blaOXA-48-like*	Escalate to tigecycline	Met—oxygenation improved
P036	*K. pneumoniae*	*blaKPC*	Escalate to carbapenem / inhibitor	Met—rapid defervescence
P041	*S. aureus*	*mecA*	Add vancomycin	Not met—switched to linezolid later
P045	*E. faecium*	*vanA*	Add linezolid	Met—AST vancomycin-R
P048	*K. pneumoniae*	*blaKPC*	Upgrade to meropenem/inhibitor	Met
P052	*E. coli*	*blaTEM*, *tetM*	Stop quinolone, continue PIP/TAZ	Met
P056	*S. aureus*	*mecA*	Vancomycin → Linezolid	Met
P060	*A. baumannii*	*blaOXA-48-like*	Tigecycline + Polymyxin B	Not met—severe comorbidities
P063	*S. aureus*	*mecA*, *ermB*	Add linezolid	Met
P068	*K. pneumoniae*	*blaCTX-M*, *sul1*	3rd-ceph → PIP/TAZ	Met
P072	*K. pneumoniae*	*blaCTX-M*	3rd-ceph → PIP/TAZ	Not met—wheezing episode
P078	*E. coli*	*qnrS*, *tetM*	Stop quinolone, add aminoglycoside	Met—age limitation

**TABLE 5:** Comparison of early clinical outcomes with or without ARG-guided therapy adjustment^*a*^

Group	Cases (*n*)	Composite endpoint achieved (*n*)	Achievement rate (%)	*χ*^2^/*P* value
ARG-guided adjustment	18	14	77.8	*χ*^2^ = 4.72, *P* = 0.030
Pathogen-guided adjustment only	12	5	41.7	

^*a*^ The “pathogen-guided adjustment only” group (*n* = 12) in the table included patients who received mNGS-guided treatment adjustments based solely on pathogen identification (without ARG information) or whose ARG results were not clinically actionable (e.g., low-confidence ARG detection). These patients were selected from the 22 mNGS-guided therapy patients excluding the 18 ARG-guided patients.

## DISCUSSION

This study evaluated the clinical utility of mNGS in diagnosing and managing severe pulmonary infections in children admitted to the PICU. Our findings demonstrate that mNGS significantly outperformed conventional microbiological methods in pathogen detection rate, spectrum breadth, mixed-infection identification, and concordance with the final clinical diagnosis, underscoring its potential as a valuable adjunct diagnostic tool for pediatric critical infections.

The pathogen detection rate achieved by mNGS (90.0%) was nearly double that of conventional techniques (48.3%), consistent with previous studies. Chen et al. reported mNGS positivity exceeding 85% in children with severe respiratory infections, far surpassing blood culture, sputum culture or PCR-based assays (22, 23). Our study further showed that mNGS could identify atypical or low-abundance pathogens, including *Brucella* spp., human metapneumovirus, and *Listeria monocytogenes*, that are typically missed by targeted or culture-dependent methods, highlighting its superior sensitivity for detecting complex infections.

Importantly, mNGS markedly improved mixed-infection detection. In this cohort, 38.8% of mNGS-positive cases involved ≥2 pathogens, compared with only 11.6% by routine testing (*P* < 0.01). Mixed infections are common in critically ill children but are often under-recognized due to the limited scope or target competition inherent in traditional assays. The unbiased, broad-spectrum nature of mNGS directly compensates for these diagnostic blind spots (24–27). Moreover, the diagnostic concordance rate between mNGS and the final clinical diagnosis reached 86.3%, far exceeding that of standard methods (55.8%). Such high alignment indicates that mNGS results more accurately reflect the true infectious etiology, providing a strong evidence base for individualized antimicrobial management.

Therapeutic outcomes in mNGS-guided patients further supported its clinical relevance. Among 46 patients with complete outcome data (selected from 89 enrolled patients after excluding those with incomplete data, transfer, or treatment withdrawal), those receiving mNGS-informed adjustments achieved earlier defervescence (77.3% vs 37.5%, *P* = 0.006), greater CRP reduction (72.7% vs 33.3%, *P* = 0.007), and improved oxygenation (77.3% vs 41.7%, *P* = 0.009) than non-guided patients. Crucially, multivariate logistic regression analysis adjusting for age, underlying diseases, PRISM III score, preoperative antibiotic use duration, and PaO_2_/FiO_2_ ratio confirmed that mNGS-guided therapy was an independent protective factor for all key outcomes (primary outcome OR = 5.23, *P* = 0.002), validating its clinical benefit beyond confounding factors. This finding confirms its capacity to optimize antimicrobial strategy in real-world critical-care settings. In the PICU, where every hour of delay can affect survival, mNGS offers a valuable second decision window, enabling evidence-based escalation, de-escalation, or regimen refinement when conventional diagnostics yield no actionable result. Recent developments in workflow optimization, such as rapid library preparation and streamlined bioinformatics, have shortened TATs to 24–36 h, making clinical integration increasingly feasible (28).

However, several challenges must still be addressed. (i) Interpretation standards remain non-uniform, with ongoing issues of background contamination and commensal interference. (ii) Clinical contextualization is essential, as detection results must be interpreted alongside host immune status, sampling site, and disease stage. (iii) Cost constraints continue to limit accessibility, particularly in resource-restricted settings.

Beyond pathogen detection, mNGS provides valuable ancillary data, including ARGs and viral subtypes, that may guide precision therapy and inform infection-control strategies. Our study innovatively linked ARG detection to clinical efficacy, showing 76.9% genotype–phenotype concordance and 77.8% composite endpoint achievement with ARG-guided therapy (vs 41.7% in pathogen-guided-only patients, *P* = 0.030). The ‘pathogen-guided adjustment only’ group included patients with mNGS-guided adjustments based solely on pathogen identification or non-actionable ARG results. The integration of mNGS data with artificial intelligence-assisted analytics is expected to further enhance diagnostic accuracy and real-time clinical decision support (19). Still, it is crucial to emphasize that mNGS should be viewed as a complement, not a replacement, for traditional microbiological methods. Its maximal clinical benefit depends on a multidisciplinary, closed-loop workflow that links laboratory detection, expert interpretation, and rapid therapeutic response.

In summary, mNGS demonstrates robust performance in pathogen identification for severe pediatric pneumonia, providing comprehensive microbiological insights that can directly inform precision antimicrobial therapy. Within the PICU setting, it surpasses conventional tests in detection rate, pathogen diversity, and co-infection recognition. Our data suggest that mNGS not only yields molecularly traceable evidence (e.g., pathogen reads and ARGs) highly consistent with phenotypic AST results but also translates into improved clinical outcomes following targeted treatment adjustments. Multivariate logistic regression confirms mNGS-guided therapy as an independent predictor of better outcomes, and ARG-informed adjustments further enhance therapeutic efficacy. The observed continuum—from molecular detection → phenotypic concordance → actionable therapy → better outcomes—illustrates that the clinical value of mNGS lies not merely in “detecting more” but in increasing the proportion of actionable, clinically relevant information.

Our study has several limitations. First, as a retrospective single-center study, the findings may be influenced by local pathogen prevalence and institutional practices. Second, the discrepancy in sample types—where mNGS primarily utilized BALF while conventional methods used various respiratory secretions—may introduce detection bias. Furthermore, although we adjusted for PRISM III score and preoperative antibiotic use duration in our regression models, residual confounding may still exist due to unmeasured variables. Finally, although mNGS offers high diagnostic value, its routine use in the PICU must be balanced against its cost; however, its potential to reduce ineffective antibiotic exposure may offer long-term cost benefits. Given the single-center retrospective design and limited sample size, potential selection bias and residual confounding cannot be ruled out. Future multicenter prospective studies with larger cohorts are warranted to validate these findings, evaluate cost-effectiveness across stratified patient groups, and establish standardized diagnostic and reporting pathways to promote the evidence-based integration of mNGS into pediatric critical-care infection management.
